# Supplementary material for: A Universal Approach to Analyzing Transmission Electron Microscopy with ImageJ
Source: Cells. 2021 Aug 24;10(9):2177. doi: 10.3390/cells10092177 (PMC8465115; doi:10.3390/cells10092177)
Supplement: Supplementary file 1 [file cells-10-02177-s001.zip › cells-1284131-supplementary file August 21 2021.pdf]

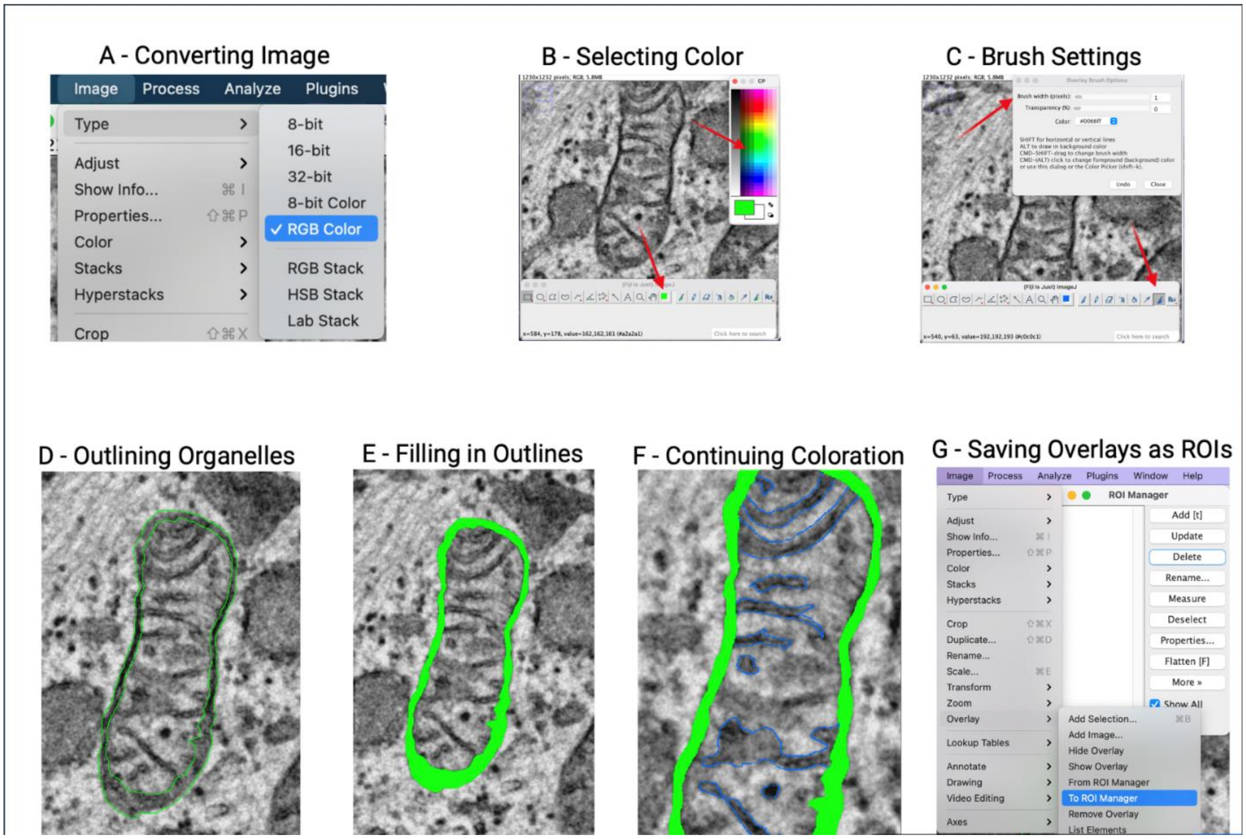

Supplementary Figure S1. Performing Pseudocoloration with Image J. (**A**) Conversion of standard format of TIFF files to RGB to allow for coloring. (**B,C**) Adjustments of the brush and color will depend on specific image used, for these representative colorations, this shows the settings used and red arrows highlight the steps to get to them. (**D**) Before filling in, outlining should be done to capture the finer details. (**E, F**) This representative image shows the coloration of the outer mitochondrion membrane (filled in, in green) and the cristae (outlined in blue, to be filled in). (**G**) Saving to ROI manager allows for the option to save the colors separately as areas that can be reported; however, given the use of it to designate organelle colors in relation to each other, this may not be needed.

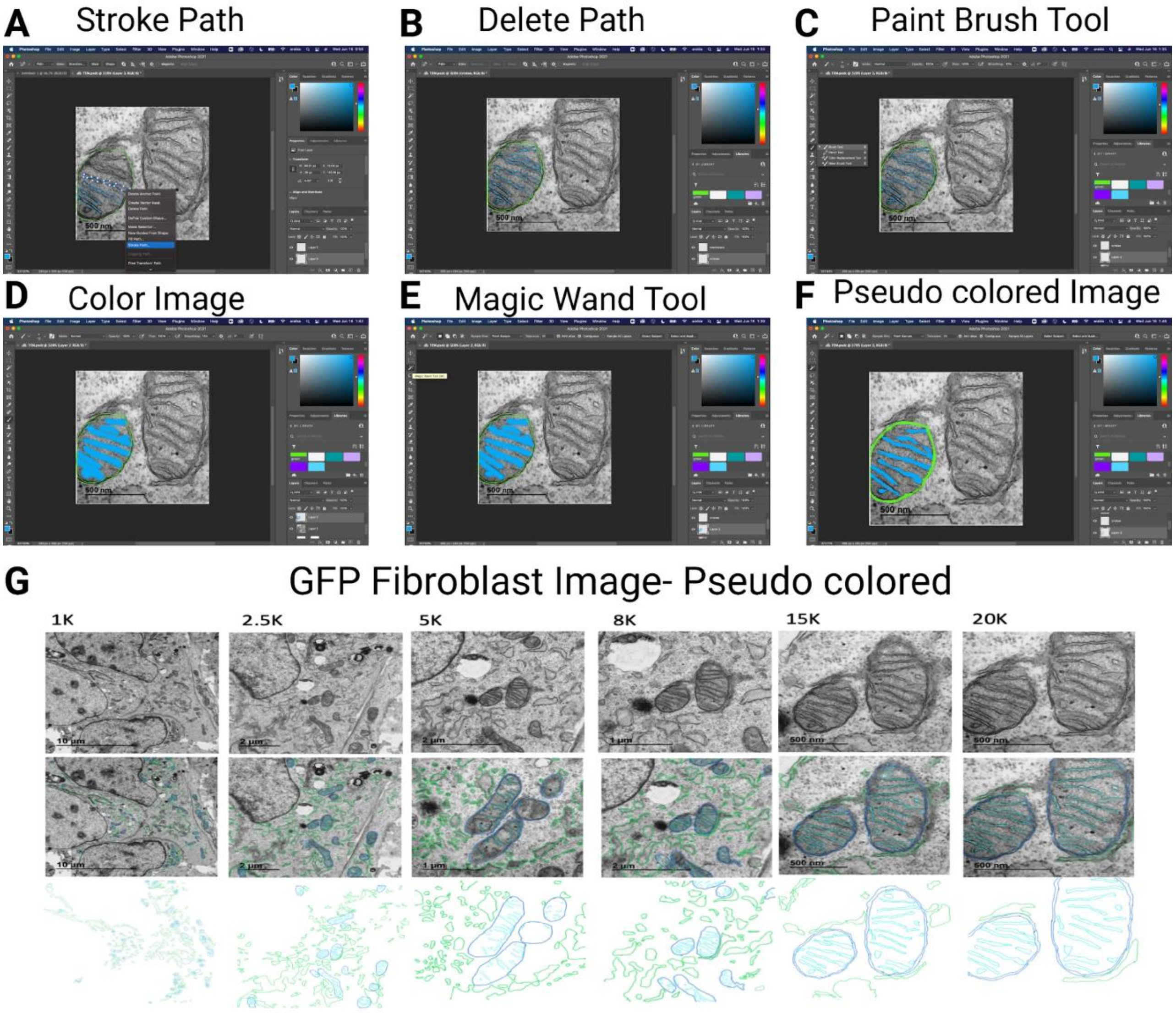

Supplementary Figure S2. Pseudocoloration of organelles with Adobe Photoshop. Organelles at various magnifications can be ported from ImageJ to Adobe Photoshop for outlining and coloration. This shows cristae segmentation and coloration with blue and mitochondria with green. Representative images are shown for each magnification to the left. Coloration allows for the clean presentation and easy visual analysis of TEM images. (**A**) Image showing the utilization of the stroke path feature in Adobe for reducing pixelated edges. (**B**) Image showing a mitochondrion that has been outlined in color in Adobe Photoshop. (**C**) Image showing selection of brush tool from the toolbar in Adobe Photoshop. (**D**) Image showing the pre-correction colored in organelle in Adobe Photoshop. The initial coloration does not need to be exact.(**E,F**) The selection and application of the Magic Wand tool fits the colors into the previously created outlines and allows for the pseudo-coloration of organelles. (**G**) The representative figure at varying magnifications of the mitochondria in fibroblast cells.
